# Peripheral Intravenous Cannula Fracture With Intravascular Retention: Early Diagnosis and Management in a Case Report and Literature Review

**DOI:** 10.7759/cureus.106602

**Published:** 2026-04-07

**Authors:** Yasser Ouatab, Marouane Jidal, Youssef Halhoul, Hassan Kraitiss, Mustapha Bensghir

**Affiliations:** 1 Department of Anesthesia and Intensive Care, Mohammed V Military Teaching Hospital, Rabat, MAR

**Keywords:** case report, catheter fracture, peripheral intravenous cannula, retained intravascular foreign body, ultrasonography (usg), venotomy

## Abstract

Fracture of a peripheral intravenous cannula with intravascular retention is a rare but potentially serious complication. The retained catheter fragment may migrate within the venous system and lead to thrombosis, infection, arrhythmia, or embolization.

We report the case of a patient in whom fracture of a peripheral intravenous cannula was suspected during routine removal, when the distal segment was found to be missing. On physical examination, a cord-like structure was palpable along the antecubital venous pathway without signs of local inflammation. Bedside ultrasonography was performed and revealed an approximately 5-cm linear intravascular fragment located within a superficial antecubital vein. Given the risk of proximal migration, urgent surgical exploration under local anesthesia was undertaken. The retained fragment was successfully retrieved through a limited venotomy without complications, and the postoperative course was uneventful.

This case highlights the importance of early recognition of catheter fracture, particularly when removal appears incomplete or difficult. Prompt localization, preferably using ultrasonography for superficial veins, is essential to guide management and prevent migration. Early surgical retrieval remains the treatment of choice for accessible fragments. Preventive measures, especially avoiding reinsertion of the introducer needle into a partially advanced catheter, are crucial to reduce the risk of this complication.

## Introduction

Peripheral intravenous catheters (PIVCs) are the most commonly used invasive devices in routine hospital care and are associated with a broad spectrum of complications, including phlebitis, infiltration/extravasation, occlusion, leakage, dislodgement, and infection [1,2]. Fracture of the catheter is distinctly uncommon but potentially serious because the retained segment becomes an intravascular foreign body that may migrate and cause thrombosis, sepsis, arrhythmia, or pulmonary/cardiac embolization [3]. Although intravascular catheter embolization has been recognized since the 1950s [4], the published literature on fractured short peripheral cannulas remains limited to isolated case reports and small case series [5-19].

The incidence of peripheral intravenous cannula fracture is extremely low and likely underreported, as most data come from isolated case reports and small case series.

Although rare, this complication may be associated with significant morbidity and, in exceptional cases, mortality, particularly when diagnosis is delayed. Furthermore, there is a lack of standardized guidelines or validated scoring systems for the management of retained PIVC fragments, highlighting the importance of early recognition and appropriate intervention.

We report a case of peripheral intravenous cannula fracture recognized at removal, with retention within the superficial antecubital venous system, and summarize representative published cases.

## Case presentation

**Figure 1:**
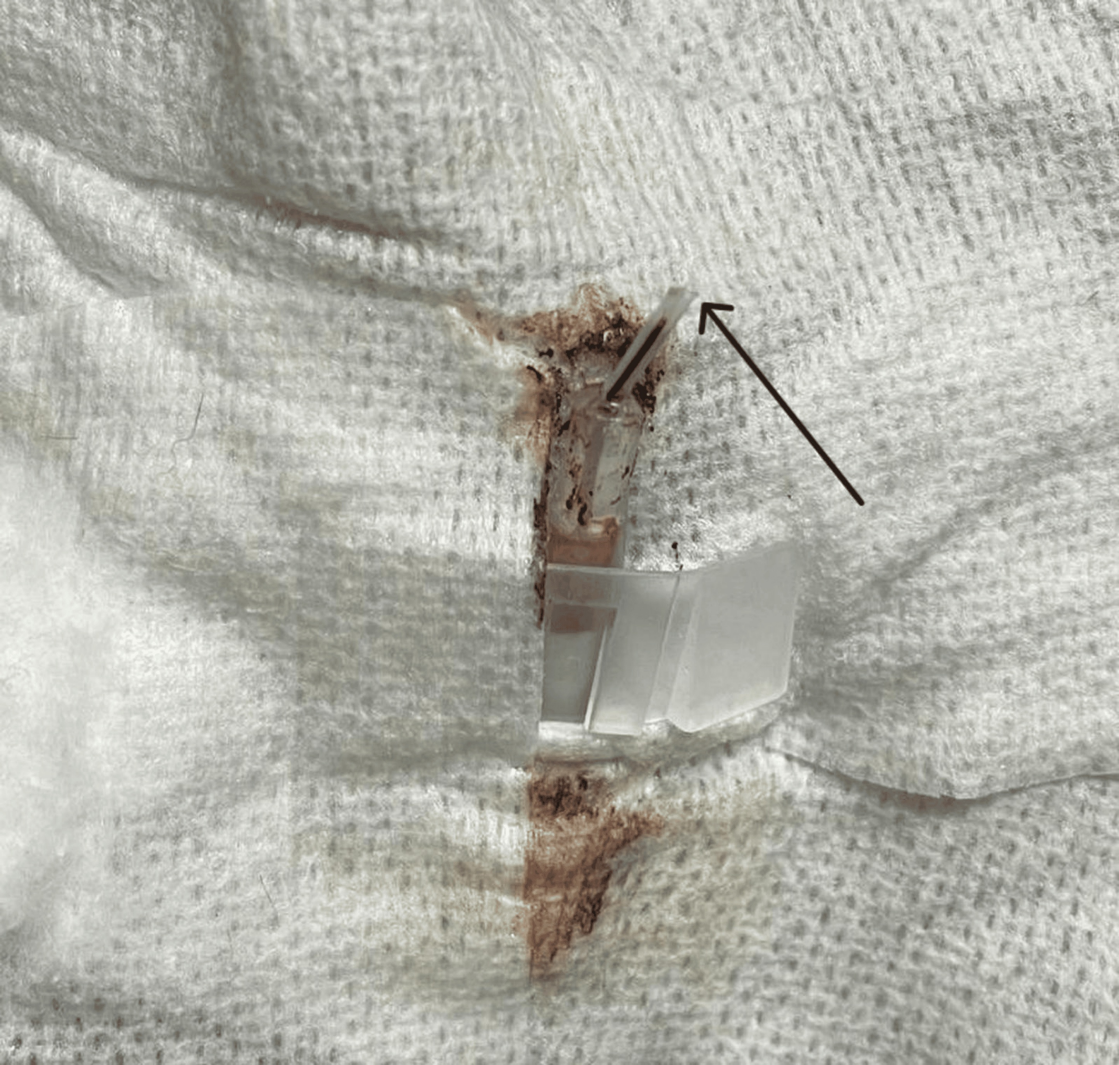
Peripheral intravenous cannula after removal The removed cannula shows loss of the distal catheter segment. The arrow indicates the missing tip, raising suspicion of intravascular retention.

During routine removal of a peripheral intravenous cannula, only part of the device was retrieved (Figure 1), raising immediate concern for catheter fracture. On physical examination, a firm cord-like structure was palpable approximately 1 cm proximal to the antecubital fossa, following the course of the superficial antecubital venous system. There were no signs of erythema, swelling, hematoma, or marked tenderness at the insertion site.

**Figure 2:**
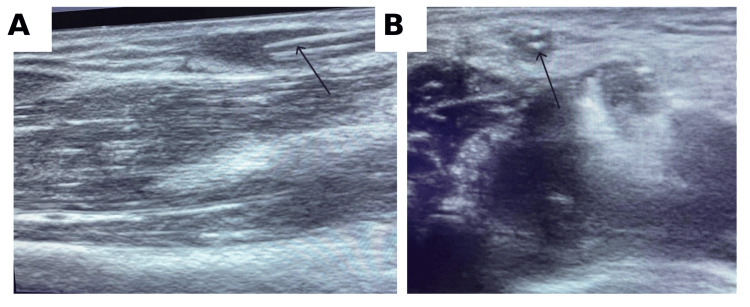
Ultrasonographic localization of the retained catheter fragment (A) Long-axis view showing a linear echogenic intraluminal structure (arrow). (B) Short-axis view confirming the retained fragment within the superficial antecubital vein (arrow).

Given suspicion of a retained intravascular fragment, bedside ultrasonography was performed to localize the foreign body and assess its depth. Sonography demonstrated an approximately 5-cm linear echogenic structure lying intraluminally within a superficial antecubital vein, consistent with the median cubital/cephalic venous pathway (Figure 2).

**Figure 3:**
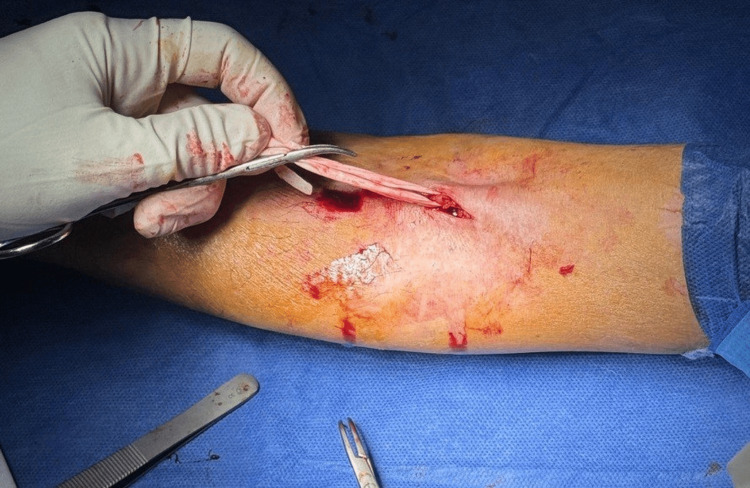
Surgical exposure of the retained catheter fragment Limited local exploration under local anesthesia shows the retained cannula segment within the superficial antecubital vein prior to extraction.

A general surgery consultation was obtained. Because the fragment was still relatively accessible and there was concern for proximal migration, urgent local exploration under local anesthesia was recommended (Figure 3).

**Figure 4:**
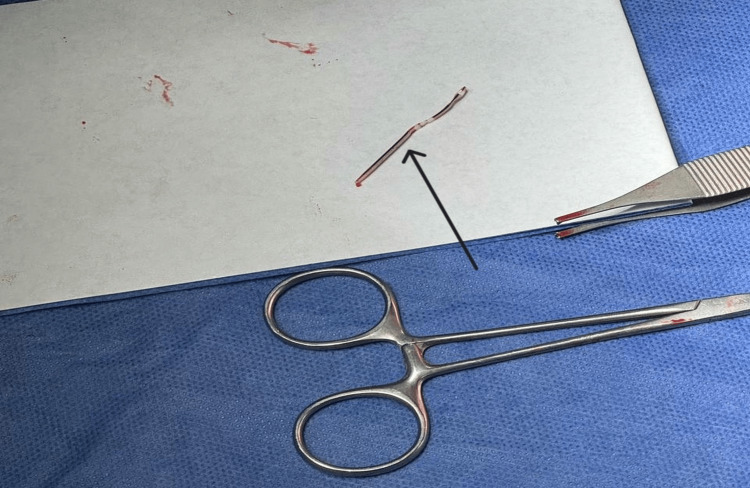
Retrieved catheter fragment The arrow indicates the distal fractured segment of the peripheral intravenous cannula after successful removal.

After informed consent, a limited venotomy was performed, and the fractured catheter segment was removed intact without complication (Figure 4).

The postoperative course was uneventful.

## Discussion

Intravascular catheter fracture and embolization are well recognized in central venous access, but they are much less frequently reported with short peripheral cannulas [3,4]. The earliest fatal intravascular catheter migration was described by Turner and Sommers in 1954 [4]. Since then, systematic reviews of catheter embolization have shown that retained intravascular fragments may remain asymptomatic or present with catheter malfunction, arrhythmia, pulmonary symptoms, septic complications, or thrombosis [3].

The peripheral IV cannula literature reveals recurring mechanisms. The most consistently implicated factor is reinsertion of the introducer needle into a partially advanced plastic catheter, which can shear or weaken the catheter wall [6,10,13]. Other reported contributors include repeated cannulation attempts using the same device, excessive manipulation, patient agitation or self-removal, difficult venous access, and repeated motion at a nearby flexion site [7,10-12,15,16,19].

Early recognition is critical. Any cannula that appears shortened after removal, any unexpected resistance during withdrawal, or any unexplained pain or swelling along the venous course should prompt immediate inspection of the catheter, limitation of limb movement, and localization of the fragment. For superficial fragments, ultrasonography is the preferred first-line imaging modality because it is rapid, nonirradiating, and useful for both localization and preoperative planning [6,11,13]. When the fragment is nonpalpable, radiolucent, deeply located, or has migrated beyond the field accessible to ultrasound, computed tomography may be helpful [5,8,12,15,19].

Most reported peripheral cases were managed successfully by early surgical retrieval via local exploration or venotomy [5-17,19]. Delayed diagnosis increases the risk of migration to more proximal veins, the pulmonary arteries, or even the heart, as illustrated by rare reports of pulmonary artery embolization and intracardiac embolization [9,18]. The present case closely parallels previously reported antecubital-vein cases in which early ultrasound localization permitted straightforward local retrieval [6,11,13].

Published reports remain sparse, but the number of case reports and small series has increased over the last decade, suggesting that this complication may be underrecognized rather than exceptional. Table 1 summarizes selected published cases and case series of peripheral intravenous cannula fracture with intravascular retention or migration.

**Table 1:** Selected published cases and case series of peripheral intravenous cannula fracture with intravascular retention or migration This table summarizes representative published reports of peripheral intravenous cannula fractures, including patient characteristics, venous site or destination of the retained fragment, suspected mechanisms, methods of detection/localization, and management with outcomes.

Reference	Patient	Venous site/destination	Suspected mechanism	Detection/localization	Management/outcome
Bloom et al. [5]	Adult man	Peripheral wrist vein → antecubital cephalic vein	Cannula separation with distal embolization	Computed tomography after proximal tourniquet	Local surgical retrieval; favorable outcome
Glassberg et al. [6]	Adult	Median cubital vein	Reinsertion of the needle into the advanced catheter	Immediate ultrasound	Venous cutdown; favorable outcome
Khadim et al. [7]	Adult	Dorsal digital vein	Repeated failed insertions using the same cannula	Radiography and computed tomography	Venotomy; favorable outcome
Singh et al. [8]	Adult	Superficial cubital vein	Fracture with local migration	Computed tomography	Phlebotomy incision and extraction; favorable outcome
Dell’Amore et al. [9]	Adult	Pulmonary artery	Delayed embolization of a peripheral catheter fragment	Thoracic imaging	Surgical retrieval; favorable outcome
Khoo et al. [10]	30-year-old woman	Dorsal metacarpal vein	Multiple attempts, reinsertion, agitation/self-removal	Radiography	Venotomy; favorable outcome
Kumar and Ranjan [11]	23-year-old woman	Cephalic vein near cubital fossa	Fracture recognized during removal	High-resolution ultrasound	Venotomy; favorable outcome
Adeosun et al. [12]	30-month-old boy	Peripheral vein/tissue retention	Multiple attempts using the same cannula	Computed tomography	Surgical removal under general anesthesia; favorable outcome
Nyamuryekung’e et al. [13]	76-year-old man	Median cubital vein	Guide-needle reinsertion	Ultrasound	Local exploration/venotomy; favorable outcome
Masood et al. [14]	Adult	Cephalic vein	Retained broken cannula	Preoperative imaging localization	Venotomy with Fogarty retrieval; favorable outcome
Chhikara et al. [15]	Adult	External jugular vein	Repeated attempts and neck movement	Non-contrast CT neck	Local removal; favorable outcome
Woo et al. [16]	63-year-old woman	Hand vein	Multiple attempts and difficult access	X-ray	Emergency department venotomy; favorable outcome
Isiguzo et al. [17]	Six adults	Peripheral veins (case series)	Various mechanisms	Clinical and imaging localization	J-flap retrieval in all cases
Wang et al. [18]	Preterm infant	Right ventricle/right atrium	Embolized peripheral catheter fragment	Echocardiography	Median sternotomy under cardiopulmonary bypass; favorable outcome
Pu et al. [19]	Neonate and infant (2 cases)	Axillary vein; superficial temporal vein	Manipulation and delayed recognition	Ultrasound ± CT angiography	Surgical retrieval; favorable outcome

This case highlights the benefit of early ultrasonographic localization and prompt surgical retrieval; however, its main limitation is the single-case design, which limits generalizability.

## Conclusions

Peripheral intravenous cannula fracture is a rare but potentially serious complication that requires early recognition and prompt management to prevent migration and adverse outcomes.
